# LMD Method and Multi-Class RWSVM of Fault Diagnosis for Rotating Machinery Using Condition Monitoring Information

**DOI:** 10.3390/s130708679

**Published:** 2013-07-05

**Authors:** Zhiwen Liu, Xuefeng Chen, Zhengjia He, Zhongjie Shen

**Affiliations:** State Key Laboratory for Manufacturing Systems Engineering, Xi'an Jiaotong University, Xi'an 710049, China; E-Mails: lzw1682007@126.com (Z.L.); hzj@mail.xjtu.edu.cn (Z.H.); zjshen.2007@stu.xjtu.edu.cn (Z.S.)

**Keywords:** local mean decomposition, reproducing wavelet kernel support vector machines, fault diagnosis, sensor-based vibration signals, rotating machinery

## Abstract

Timely and accurate condition monitoring and fault diagnosis of rotating machinery are very important to maintain a high degree of availability, reliability and operational safety. This paper presents a novel intelligent method based on local mean decomposition (LMD) and multi-class reproducing wavelet support vector machines (RWSVM), which is applied to diagnose rotating machinery faults. First, the sensor-based vibration signals measured from the rotating machinery are preprocessed by the LMD method and product functions (PFs) are produced. Second, statistic features are extracted to acquire more fault characteristic information from the sensitive PF. Finally, these features are fed into a multi-class RWSVM to identify the rotating machinery health conditions. The experimental results validate the effectiveness of the proposed RWSVM method in identifying rotating machinery fault patterns accurately and effectively and its superiority over that based on the general SVM.

## 1. Introduction

Rotating machinery fault diagnosis is actually a pattern recognition process [1,2], which includes acquiring the information, extracting the features and recognizing the conditions. The latter two are the key links.

The purpose of extracting the features is to extract parameters representing the machine operation conditions to be used for machine condition identification. There are a number of feature extraction methods for vibration signals in the literature. A popular and noted example is the time-frequency analysis method of wavelet transform, which has obtained great success in machine fault diagnostics for its many distinct advantages [3,4]. However, wavelet transform, is essentially an adjustable window Fourier transformation. On the one hand, due to the limited length of the wavelet base function, energy leakage would occur inevitably in a wavelet transformation [5]. In addition, the result of a wavelet transform depends on the choice of the wavelet basis function, and only those signal features that match well with the shape of the wavelet basis function have a chance to be detected, while all other features will be masked or even completely ignored. Therefore, wavelet transform is not a self-adaptive signal processing method in nature [6]. Empirical Mode Decomposition (EMD) proposed by Huang *et al.* [5], is a self-adaptive signal processing method that could decompose a complicated signal into a number of intrinsic mode functions (IMFs). Unlike the wavelet transform, EMD has no need for a basis function and no need for a Fourier transformation, and can perform decomposition of the raw signal and automatically determine the level of decomposition based on the nature of that raw signal. Frequency components contained in each IMF not only relate to the sampling frequency, but also change with the signal itself. Furthermore, the whole transform process would not lead to energy diffusion and leakage. Fault diagnosis based on the EMD has been improved and successfully applied to rotating machines [7–9].

Local mean decomposition (LMD) is a novel adaptive time–frequency analysis method proposed by Smith [10] in 2005. It is suitable for the analysis of multi-component non-linear and non-stationary signals caused by faults in rotating machinery [11–13]. Moreover, the differences between the two adaptive methods LMD and EMD are given in [14] where it was shown that LMD is superior to the EMD method in four aspects. Therefore, the LMD technique is further investigated to preprocess the vibration signals to highlight the features in this work.

Final condition identification is another task in fault diagnosis of rotating machinery. Machine condition identification via artificial intelligence techniques can provide an automated fault diagnosis procedure [15,16]. However, lack of fault samples is the main bottleneck. Support vector machine (SVM) developed by Vapnik in 1999 is an effective method for pattern recognition with small samples [17]. The basic idea of SVM is to implement nonlinear transformations by defining an appropriate kernel function. The input space is transformed into a high-dimensional space and then the optimal liner classification hyper-plane is calculated in the new space [18], so the kernel function plays a very important role in the SVM method. However, due to the limitations of different kernel functions and the difficulty of designing a universal kernel function, novel kernel functions still need be studied, designed and applied to SVM.

The combination of wavelet analysis and SVM kernel function is a new idea and technology, which has the advantages of better accuracy, generalization capability and multi-resolution [19]. In this paper, we present a novel support vector classification model based on reproducing wavelet support vector machines (RWSVM), which is an expansion and improvement of the general SVM. RWSVM model is based on the theory of wavelet analysis and Reproducing Kernel Hilbert Space (RKHS) [20]. A new reproducing wavelet kernel is constructed by wavelet basis function in different resolution, which is used to construct support vector classification model.

To automatically and effectively diagnose rotating machinery faults, a novel fault diagnosis method based on LMD and RWSVM is proposed in this paper. In the proposed method, the sensor-based vibration signals captured from the rotating machinery are first decomposed by the LMD method. Second, the most sensitive PF that contains the main fault information is selected. Third, statistic features are extracted from the most sensitive PF. Finally, these features are input into the RWSVM to recognize the health conditions of the rotating machinery. The identification results validate the effectiveness of the proposed method.

## 2. The Proposed Method

### 2.1. LMD Algorithm

LMD was originally developed to decompose modulated signals into a small set of product functions (PFs), each of which is the product of an amplitude envelope signal and a frequency modulated (FM) signal. Different from EMD, the essence of the LMD method is to isolate pure FM signals and envelope signals from the original signal by iteration, and then multiply the pure FM signals with envelope signals to get a PF component whose instantaneous frequency is physically meaningful.

A more detailed and comprehensive explanation of LMD is provided in references [11,14]. After a series of calculations, the original signals are decomposed as follows:

(1) $$x(t)=\sum_{i=1}^{n}PF_{i}(t)+{\text{res}}_{n}(t)$$

where *x*(*t*) is the original signal, *res_n_*(*t*) is the residue.

**Figure 1.**
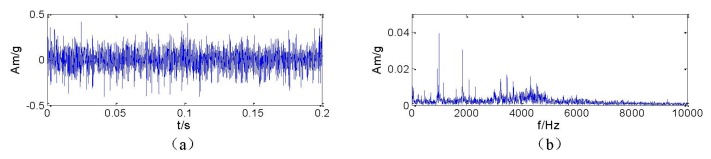
The time domain waveform and spectrum of the vibration acceleration signal of the rolling bearing with localized defects on the outer race: (**a**) time domain waveform and (**b**) spectrum.

In order to verify the effectiveness of the proposed signal transient detection method, an application example for the detection of localized outer-race defects of rolling bearing (type ZA-2115) is provided here. The rotating frequency *f_r_* of the shaft is 66.66 Hz. Based on the geometric parameters and rotating frequency [21], the characteristic frequency of the outer-race defect is 236.4 Hz. The sampling frequency is 20 kHz. Figure 1 shows the time domain waveform and spectrum of the vibration acceleration signal. It can be observed that the fault feature is so weak that it is drowned by the background signals related to the rotary speed of the rotor and other noise.

**Figure 2.**
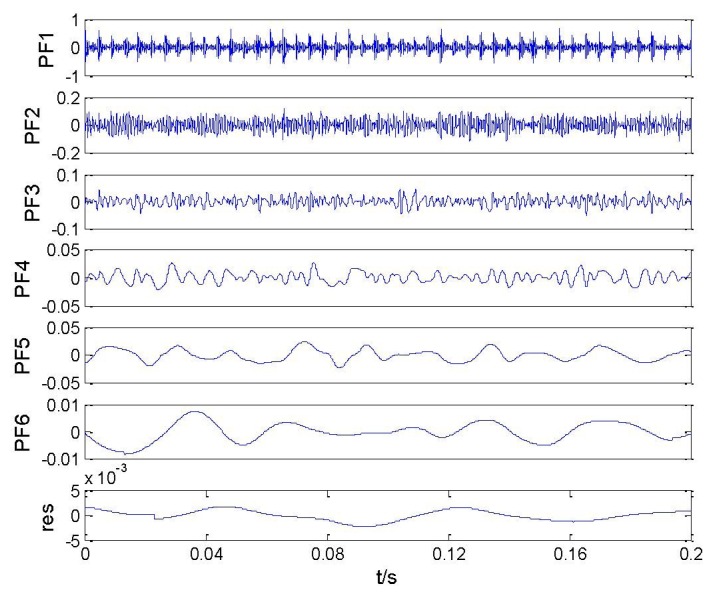
The decomposed results and residue by LMD of a bearing outer race fault signal.

**Figure 3.**
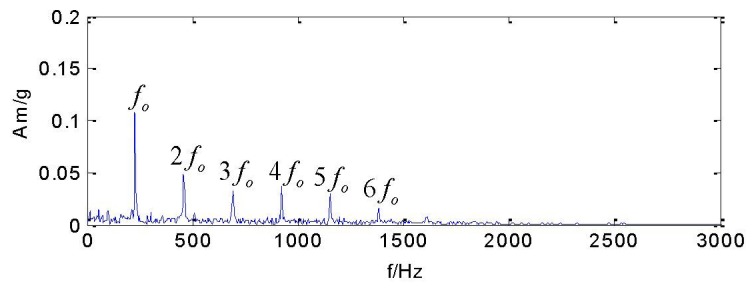
Frequency spectrum of PF1.

The LMD is employed to decompose the vibration acceleration signal and a total of six PFs and the residual item are obtained, as shown in Figure 2. It can be clearly distinguished in this figure that there are obvious periodical impulses in the time domain in PF1. The frequency spectrum analysis of this signal is shown in Figure 3. The fault characteristic frequency of the outer race, *f_o_*, and its harmonics (2*f_o_*, 3*f_o_*, 4*f_o_*, 5*f_o_*, 6*f_o_*) can be clearly observed.

As shown in Figure 3, it is clear that the rolling bearing fault frequency feature can be obviously distinguished. Therefore, the LMD method is effective and available in analyzing those vibration signals of rotating machinery which usually have modulated characteristics.

### 2.2. Multi-Class RWSVM

The main idea in this proposed method is to construct a reproducing wavelet estimator by solving an empirical risk minimization problem. According to the learning theory [22], learning from samples can be viewed as the classification of the functional dependency between an input *x* and an output *y* of a system given a set of examples:

(2) $$S=\left\{\left(x_{i},y_{i}\right){|x_{i}\in x\subset R}^{N},y_{i}\in y\subset \left\{-1,1\right\},i=1,\cdots ,l\right\}$$

In order to avoid ill-posed problems, we have to look for the function f that minimizes the regularized empirical risk functional instead of the empirical risk according to regularization theory [23].

(3) $$R_{\text{reg}}[f]=\frac{1}{l}\sum_{i=1}^{l}C(x_{i},y_{i},f(x_{i}))+\lambda {\left\|f\right\|}_{H}^{2}$$

Where C(·,·) is a cost function, *H* is a Reproducing Kernel Hilbert Space (RKHS) and *λ* is a regularization parameter.

By minimizing the regularized empirical risk in Equation (3), RWSVM estimator has the following form:

(4) $$f(x)=\text{sign}\left(\sum_{i=1}^{l}\alpha_{i}y_{i}K(x,x_{i})+b\right)$$

Where b ∈ *R*, α*_i_* is the Lagrange multiplier, and *K*(·,·) is the reproducing wavelet kernel function with the form:

(5) $$K\left(x_{i},x_{j}\right)=\langle \Phi^{T}\left(x_{i}\right)\cdot \Phi \left(x_{j}\right)\rangle ,\;i,j=1,\ldots ,l$$

In the case of RWSVM method, *K*(·,·) is a multi-scale wavelet kernel constructed in the RKHS. RKHS is a Hilbert space with special properties. The interest of RKHS arises from its associated kernel functions. In learning theory, some advance of kernel issue has shown the importance of using a wisely chosen kernel in RKHS as it largely influences the generalization capability [24].

#### Theorem

Wavelet frames' finite set of *L*^2^(R) is a Hilbert space endowed with inner product spans a RKHS. Let's define an indexed family of function Γ*_t_* ∈ *L*^2^(R) index by t ∈ *x* (*x* being any subset of *R^n^*), its reproducing kernel is *K*(*x*,*y*) = <Γ*_x_*(·), Γ*_y_*(·) > *L*^2^(*R*). Interested readers should refer to [25,26] for details.

Consider the family *e_i_*(·) as an orthonormal basis of *L*^2^(*R*) and let *Φ_i_* be a point-wise defined wavelet basis of *L*^2^(*R*). One can write:

(6) $$\Gamma_{x}(\cdot )=\sum_{i,j}\alpha_{i,j}\Phi_{j}(x)e_{i}(\cdot )$$

where α*_i,j_*=*c_j_*δ*_i,j_* is the coefficients combining the orthonormal basis {*e_i_*} of *L*^2^(R) with wavelet basis *Φ_j_* of *L*^2^(R), and *c_j_* is a coefficient depending on the considered wavelet *Φ_j_*.

So far, we can construct a wavelet kernel in RKHS as follows:

(7) $$K(x,y)=\sum_{i,j,n}\alpha_{i,j}\alpha_{j,n}\Phi_{j}(x)\Phi_{n}(y)$$

For a common multidimensional wavelet function, the mother wavelet can be given as the product of one-dimensional (1-D) wavelet function according to the tensor products of RKHS [27]:

(8) $$K_{d}(x,y)=\prod_{i=1}^{d}k(x_{i},y_{i})$$

Let ψ(*x*) be a mother wavelet and let *a* and *b* denote the dilation and translation factor, respectively, *a*, *b* ∈ *R*, then according to wavelet theory:

(09) $$\phi_{i}(x)=\psi_{j,k}(x)=\frac{1}{\sqrt{a_{0}^{j}}}\left|\psi \frac{x-kb_{0}a_{0}^{j}}{a_{0}^{j}}\right|={\left|a_{0}\right|}^{-j/2}\psi (a_{0}^{j}x-kb_{0})$$

where *a*_0_, *b*_0_ ∈ *R*, *j*, *k* ∈ *Z*, *i* denote a multi index. It is know that when the function ψ(*x*) satisfies the necessary condition (admissibility of the mother wavelet and suitable parameters with *a*_0_, *b*_0_ such as *a*_0_ = 2, *b*_0_ = 1) will lead to wavelet frames.

For practical kernel construction, we have to define a mother wavelet function ψ and select suitable parameters according to the problem at hand. Moreover, we can truncate the range of the scales and set coefficients in Equation (7) so that the kernel in RKHS can be written as follows:

(10) $$K(x,y)=\sum_{j=j_{\min}}^{j_{\max}}\sum_{k=k_{\min}}^{k_{\max}}\frac{1}{2^{j}}\psi_{j,k}(x)\psi_{j,k}(y)$$

where *j*, *k* are the dilation and translation parameters of a mother wavelet function respectively, *j*_min_ and *j*_max_ are the minimum and maximal dilations [20], and *k*_min_ and *k*_max_ are the minimum and maximal translation of the wavelet kernel, respectively. The minimal and maximal dilations can be selected by the cross-validation model [28].

**Figure 4.**
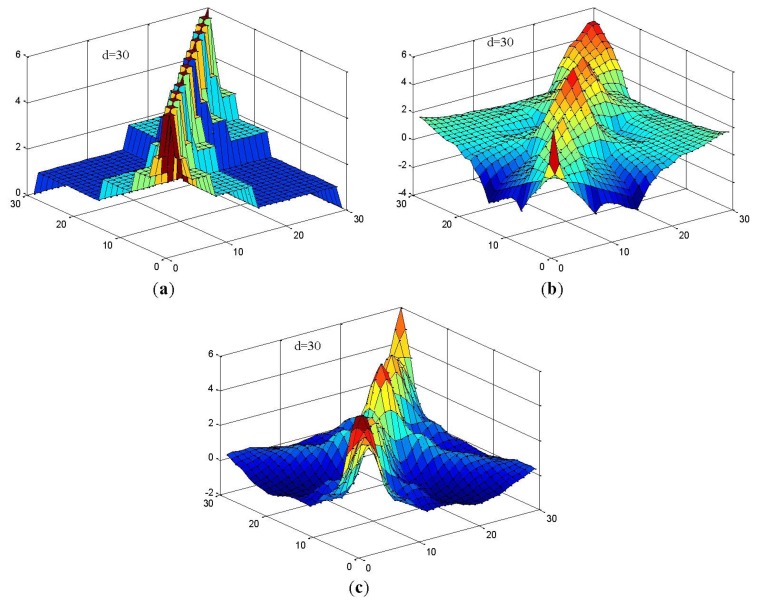
Examples of wavelet kernel: (**a**) Harr Kernel (**b**) Daubechies Kernel (**c**) Coiflet Kernel.

In this study, we constructed RWSVM with different wavelet functions. Figure 4 shows the representations of the wavelet kernel SVM function using Haar, Daubechies, and Coiflet.

**Figure 5.**
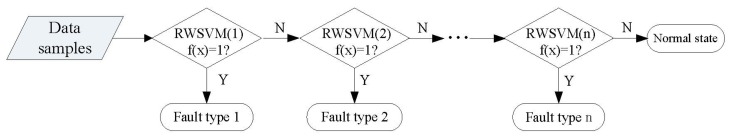
‘One-versus-rest’ multi-class RWSVM.

The initial SVM is an essentially binary classifier, and Lingras and Butz [29] developed the one-versus-rest (1-v-r) SVM for multi-classification to improve the applicability of SVM. So the multi-class RWSVM is constructed according to 1-v-r multi-class SVM. Therefore, the process of the multi-class RWSVM is described in Figure 5.

## 3. The Proposed Method

**Figure 6.**
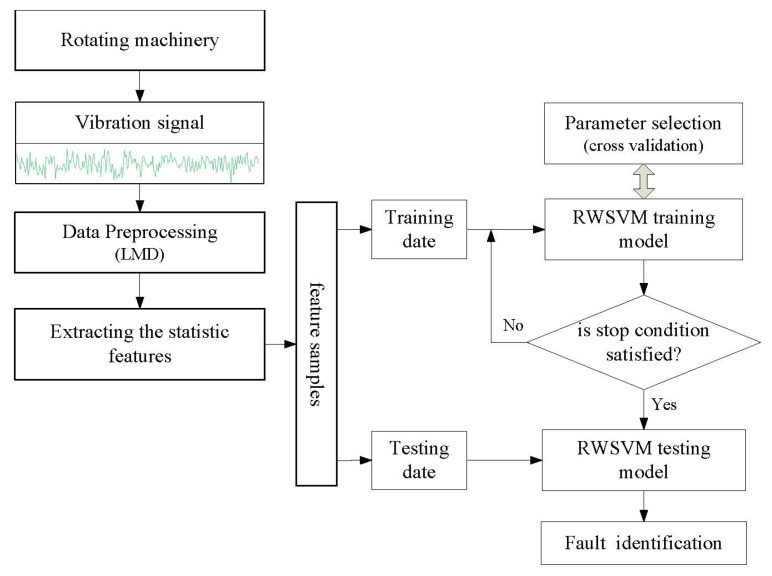
Architecture of the proposed fault diagnosis system.

A novel intelligent fault diagnosis strategy is proposed in this study, which is based on LMD and multi-class RWSVM. Figure 6 shows the architecture of the proposed fault diagnosis method. Procedure of the proposed system can be summarized as follows:

Step 1 acquiring vibration acceleration signals when the rotating machinery operation state is normal or faulty.
Step 2 preprocessing vibration signals by using LMD.
Step 3 extracting seven statistic features [30,31] (*i.e.*, peak value, mean, standard deviation, root mean square, shape factor, skewness, kurtosis, crest factor, K factor, and pulse index) by using the first three PF(the most important information of the vibration signal is included in high-frequency bands are shown in Figure 2). All features are taken as samples that are divided into two subsets, the training samples and testing samples.
Step 4 constructing classification process for fault diagnosis by RWSVM using different reproducing wavelet kernel function.
Step 5 the testing samples can be in put into the trained RWSVM classifier and then the operating conditions can be identified by the output of the RWSVM classifiers.

## 4. Experiments, Results and Discussion

In order to evaluate the effectiveness of the proposed method, two kinds of experimental setups are constructed to offer the vibration signals from various fault conditions.

### 4.1. Case 1: Gearbox Fault Diagnosis

#### 4.1.1. Experimental Data

**Figure 7.**
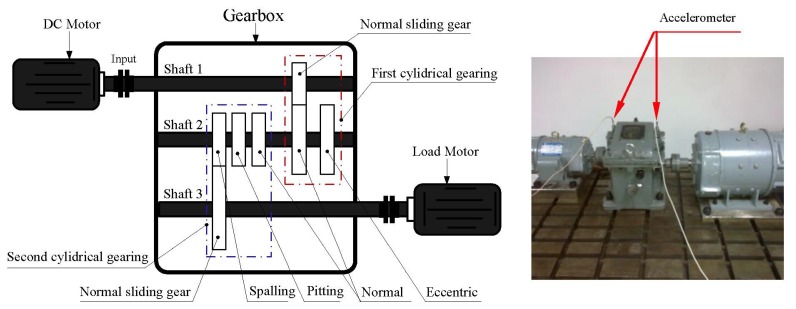
Structure sketch of the test bench for the experimental gearbox.

Figure 7 shows the structure diagram of the test bench with the gearbox faults, which consists of a DC motor, load motor, DC speed load system, gear reducer, *etc*. There are two-stage cylindrical gearings and seven gears in the gear reducer. The parameters of the gear transmission system are listed in Table 1. In the first cylindrical gearing, there are one driving gear and two driven gears. The two driven gears both have 64 teeth in axis II, and the inner one is normal while the outer is eccentric. The second gearing contains three driving gears and one driven gear. All the driving gears have 85 teeth with the condition of spalling, pitting and normality from left to right. The vibration signals are obtained with the sample frequency of 6,400 Hz when the speed of the DC motor is 1,000 rpm. The data recorder is equipped with low-pass filters at the input stage for anti-aliasing. The acceleration sensor is fixed on bearing pedestal of axle II.

**Figure 8.**
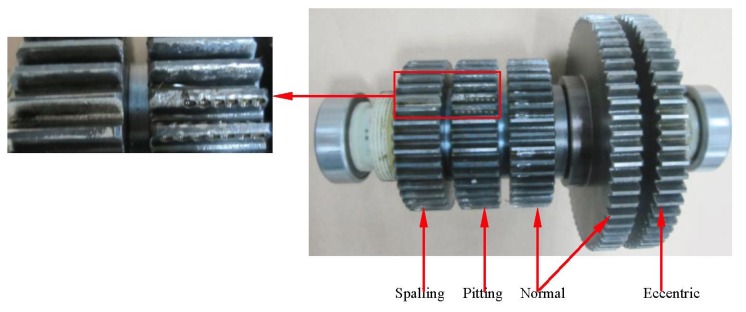
Gears defect in the shaft 2.

**Figure 9.**
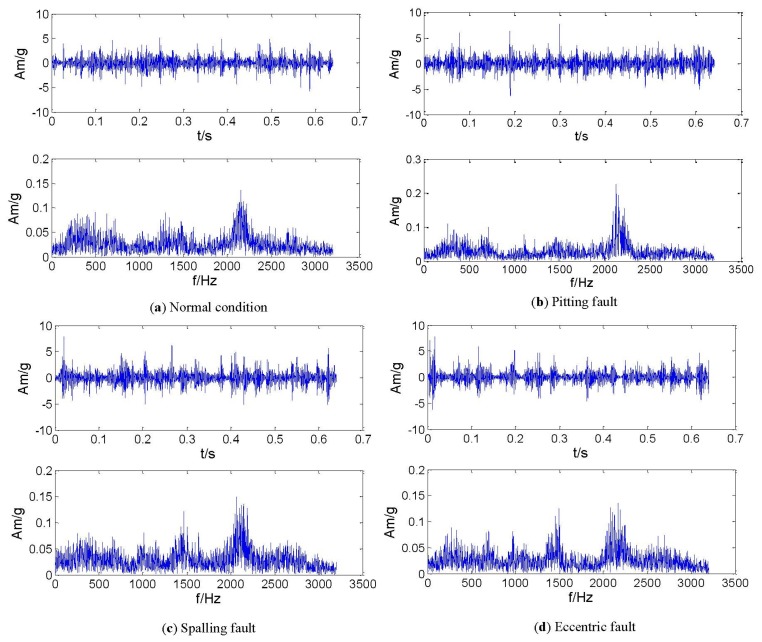
Vibration signals of time- and frequency domain.

In this experiment, a gear data set in axle II shown in Figure 8 consisting of the four conditions was obtained such as normal, pitting, spalling and eccentric (with labels 1, 2, 3 and 4, respectively). The vibration signals in the time- and frequency domain for all the gear running conditions are shown in Figure 9. For each condition, 50 samples were used, and therefore the whole data set corresponding to the four signal conditions includes 200 samples. Each sample is a section of vibration signal containing 4,096 sampling points. The whole data set is split into two sets: 120 samples for training and 80 samples for testing. The twenty-one features extracted by means mentioned in Section 3, these features are selected and input into the RWSVM classifiers to automatically identify health conditions of gear box.

#### 4.1.2. Experimental Results

**Figure 10.**
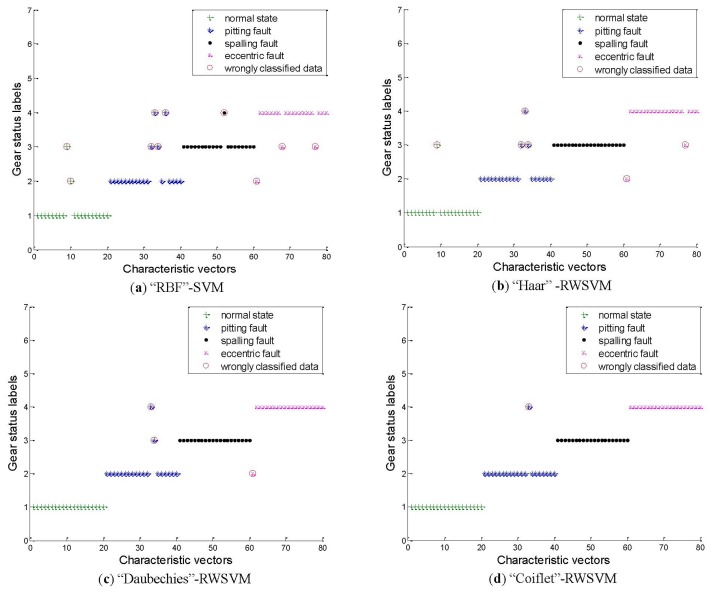
Results of the SVM classification for the experimental gear.

The present study chooses SVM with Gaussian radial basis function (RBF) kernel as a reference which is commonly preferred to other kernel function types [32,33]. The analysis results are displayed in Figure 10 and Table 2.

For the SVM using RBF kernel in Figure 10a, the classification accuracies for the normal state, pitting, spalling and eccentric fault are 90%, 80%, 95% and 85% respectively. The overall average classification accuracy is 87.5%.

As Figure 10b shows, by using the RWSVM with Harr wavelet kernel, the classification accuracies for the normal state, pitting fault, spalling fault and eccentric fault are 95%, 85%, 100% and 90% respectively. The overall average classification accuracy is 92.5%.

From Figure 10c, we can see that the RWSVM using the Daubechies wavelet kernel gives the highest classification accuracy (100%) for the normal state and spalling fault. The classification accuracies of the pitting and eccentric fault are 90% and 95% respectively. The overall average classification accuracy is 96.25%.

In Figure 10d, the RWSVM using Coiflet wavelet kernel gives the highest classification accuracy (100%) for the normal state, spalling and eccentric fault. The classification accuracy for the pitting fault is 95%. The overall average classification accuracy is 98.75%.

Furthermore, Table 2 compares the accuracy of SVM and RWSVM. Compared with the SVM using RBF kernel, the proposed methods based on RWSVM using the Harr wavelet kernel, Daubechies wavelet kernel, and Coiflet wavelet kernel improve the classification accuracy by 5%, 8.75% and 11.25%, respectively.

### 4.2. Case 2: Aero-Engine Rotor Fault Diagnosis

#### 4.2.1. Experimental Data

**Figure 11.**
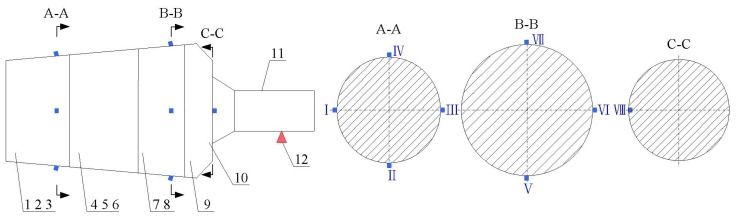
The structure of the aero-engine rotor and locations of the sensors: **1**–**9**: the first-ninth disk, **10**: shaft, **11**: location of the exciter, A-A, B-B, C-C: sections of sensor locations, I – VIII: locations of the accelerometers.

The tested aero-engine rotor system is a dismountable disk-drum type rotor whose structure sketch is shown in Figure 11. The rotor is mainly constituted of some disks, a drum and a shaft. There are 24 jointing bolts for connecting the disks with the drum in the rotor structure. Faulty is simulated by acting on these jointing bolts.

**Figure 12.**
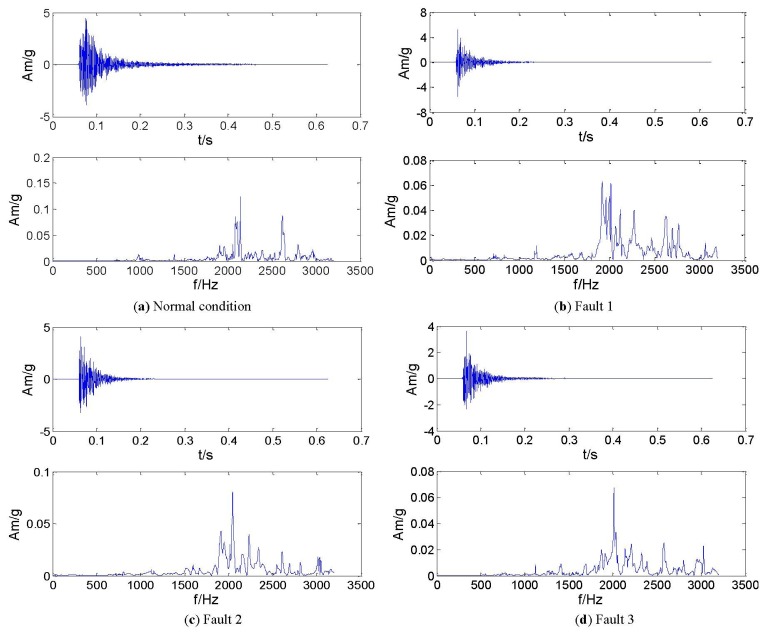
Vibration signals of time- and frequency domain.

The rotor structure is excited by utilizing a shaker with a constant excitation frequency of 1 Hz and the excitation position is on the shaft, as shown in Figure 11. For monitoring the structural response signals, eight accelerometer sensors are mounted at the location 1-8 on the rotor. A Sony EX data acquisition system is used to record the response signals and the acquisition frequency is set to 6,400 Hz. The response signals in the time- and frequency domain for all the structural states (normal and faults) are shown in Figure 12.

Each data subset consists of 50 samples and therefore the whole data set corresponding to the four bearing health conditions includes 200 samples. Each sample is a section of vibration signal containing 2,048 sampling points. Each sub-sample data set is split into two sets: 30 sub-samples for training and 20 sub-samples for testing.

#### 4.2.2. Experimental Results

**Figure 13.**
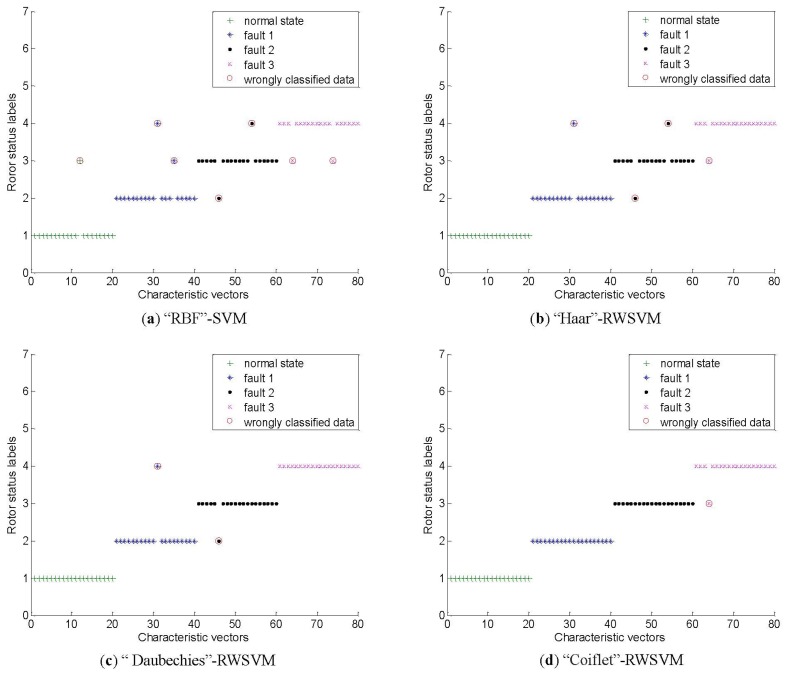
Results of the SVM classification for the aero-engine rotor.

The above-mentioned intelligent method is employed again and the corresponding identification results are given in Figure 13 and Table 3. Figure 13a–d present the testing results of SVM based on RBF kernel, and our RWSVM based on Harr wavelet kernel, Daubechies wavelet kernel, and Coiflet wavelet kernel respectively. From Table 3, it can be seen that the testing accuracies of these methods are 91.25%, 95%, 97.5% and 98.75%, respectively. Compared with the average accuracy of SVM, the proposed RWSVM method increases the recognition accuracy by 3.75%, 6.25% and 7.5% respectively.

### 4.3. Discussion

In the two experiments, test results verify that the proposed RWSVM method obviously outperforms the SVM method in diagnosing different categories of gear and aero-engine rotor faults. In two experimental results, the proposed reproducing wavelet kernels function with multi-resolution structure have a better performance and generalization ability than the traditional RBF kernel, and the best classification accuracy was obtained using the Coiflet kernel function. The main reasons are as follows:

(1) The RBF is a kind of kernel function which is generally used. It shows the good generalization ability. However, With the RBF kernel functions, the SVM can not approach any curve in *L*^2^(*R^n^*) space (quadratic continuous integral space), because the kernel function which is used now is not the complete orthonormal base. This characteristics result in that the classification SVM can not approach every classification interface in the *L*^2^(*R^n^*) space.
(2) The kernel functions of Haar wavelet, the Daubechies wavelet and the Coiflet wavelet are the orthonormal base of *L*^2^(*R^n^*) space through its dilation and translation. These kernel function can approach almost any classification interface in *L*^2^(*R^n^*) space, thus they enhance the generalization ability of the SVM. Meanwhile, reproducing wavelet kernel with multi-scale structure is not only orthonormal (or approximately orthonormal) but also suitable for local signal analysis and signal-noise separation.
(3) The Coiflet kernel function has some interesting properties that make it useful in signal processing. It possesses maximal number of vanishing shifted scaling moments for the given number of scaling coefficients. Coiflet are separable filters in the sense that spatial frequencies in *x*, *y*, and diagonal directions can be selected using this type of filters. Coiflet filters maintain a close match between the trend values of the signal and the original signal. Besides, Haar wavelet does not have the property of continuity, Coiflet wavelet has superior symmetry than that of Daubehcies wavelet.

## 5. Conclusions

The present study proposes a novel hybrid intelligent multi-fault classification method based on LMD and multi-class RWSVM. In the proposed method, LMD can select frequency bands adaptively according to the characteristics of the vibration signal and determine signal resolutions of different frequency bands. It can optimize the signal analysis and increase the accuracy of useful information extraction. RWSVM is effective in handling uncertain data and small samples, the experiment results demonstrate that RWSVM produces an obvious improvement in recognition accuracy and provides a good diagnosis capability. Compared with the general RBF-SVM method, the proposed RWSVM has better generalization ability and strong robustness.

In addition, it should be noted that although the proposed method is only demonstrated by using the gearbox and the rotor examples in this work, it can be easily applied to other classification problems in mechanical fault diagnosis. The proposed RWSVM might provide a new opportunity for other condition monitoring and fault diagnosis which still needs to be further explored in the future.

## Figures and Tables

**Table 1.** Parameters of gear transmission system.

**Total Ratio**	**First Cylindrical Gearing**			**Second Cylindrical Gearing**		
	
	Modules (mm)	The number of teeth		Modules (mm)	The number of teeth	
5.23	2	Z1	26	2	Z1	40
		Z2	64		Z2	85

**Table 2.** The classified result of gear transmission system test data.

**Operating Condition**	**Accuracy (%)**			

	SVM	RWSVM		
	
	$\text{RBF}\;\left(\begin{array}{l} c=100\\ \sigma =7 \end{array}\right)$	$\text{Haar}\;\left(\begin{array}{l} j_{\max}=6\\ j_{\min}=-6 \end{array}\right)$	$\text{Daubechies}\;\left(\begin{array}{l} j_{\max}=8\\ j_{\min}=-8 \end{array}\right)$	$\text{Coiflet}\;\left(\begin{array}{l} j_{\max}=4\\ j_{\min}=-4 \end{array}\right)$
Normal state	90	95	100	100
Pitting fault	80	85	90	95
Spalling fault	95	100	100	100
Eccentric fault	85	90	95	100

Average accuracy (%)	87.5	92.5	96.25	98.75

**Table 3.** The classified result of aero-engine rotor test data.

**Operating Condition**	**Accuracy (%)**			

	SVM	RWSVM		
	
	$\text{RBF}\;\left(\begin{array}{l} c=100\\ \sigma =5 \end{array}\right)$	$\text{Haar}\;\left(\begin{array}{l} j_{\max}=6\\ j_{\min}=-6 \end{array}\right)$	$\text{Daubechies}\;\left(\begin{array}{l} j_{\max}=4\\ j_{\min}=-4 \end{array}\right)$	$\text{Coiflet}\;\left(\begin{array}{l} j_{\max}=2\\ j_{\min}=-2 \end{array}\right)$
Normal state	95	100	100	100
Pitting fault	90	90	95	100
Spalling fault	90	90	95	100
Eccentric fault	90	95	100	95

Average accuracy (%)	91.25	95	97.5	98.75
